# Healthcare professionals' perspectives on expanding newborn genomic screening in the emirate of Abu Dhabi, United Arab Emirates: insights from a cross-sectional study

**DOI:** 10.3389/fpubh.2026.1769908

**Published:** 2026-06-03

**Authors:** Aminu S. Abdullahi, Y. M. Abu Alqumboz, Ahmed Jamal Bek, Mohammed Shaidul, Maryam Alktifan, Fatma Alhammadi, Alia Rashed Manea Alshamsi, Meera Saeed Nhayah Alkaabi, Marwa Alkatheeri, Taif AlEissaee, Mohammad Alsaadi, Mohammedfaeq Alqolaq, Omar Alzaabi, Khalid Almaamari, Nasser Abdulla Alshamsi, Saoud AlTamimi, Khalifa Alseiari, Ahmed AlQedra, Mohamed Salem Alameri, Azhar T. Rahma

**Affiliations:** 1 Institute of Public Health, College of Medicine and Health Sciences, United Arab Emirates University, Al Ain, United Arab Emirates; 2 Burjeel Royal Hospital, Al Ain, United Arab Emirates; 3 Department of Internal Medicine, Zulekha Hospital, Dubai, United Arab Emirates; 4 Department of Health, Abu Dhabi, United Arab Emirates

**Keywords:** newborn screening, genomic literacy, healthcare professionals, ethical considerations, tandem mass spectrometry, United Arab Emirates, attitudes

## Abstract

**Background:**

Genomic Newborn Screening (gNBS) could transform neonatal healthcare by enabling early detection of genetic disorders, timely intervention, and improved health outcomes. This study assessed healthcare professionals' self-reported knowledge, attitudes, and challenges regarding expanded newborn genomic screening in Abu Dhabi, United Arab Emirates (UAE), and identified factors influencing its integration into neonatal healthcare.

**Methods:**

A cross-sectional survey was distributed to healthcare professionals across public and private facilities in Abu Dhabi, using random and snowball sampling. Data were analyzed using descriptive statistics, chi-square tests, and logistic regression to examine associations between gNBS self-reported knowledge, attitudes, and professional characteristics.

**Results:**

About 46% (95% CI: 40%−52%) of professionals demonstrated good self-reported knowledge of gNBS, with significantly higher awareness among those experienced in newborn screening and tandem mass spectrometry. While 70% supported expanding gNBS, key barriers included cost, lack of screening guidelines, and ethical concerns such as genetic privacy and psychological distress for families. Most participants (85%) supported upskilling nurses and midwives for cord blood collection. Additionally, severe combined immunodeficiency (SCID), Duchenne muscular dystrophy (DMD), and spinal muscular atrophy (SMA) were essential conditions for inclusion in expanded gNBS programs.

**Conclusion:**

Healthcare professionals in Abu Dhabi recognize the potential of gNBS but also acknowledge key implementation challenges. Advancing implementation requires training, policy development, and ethical considerations. Future efforts should prioritize cost-effectiveness evaluations, regulatory frameworks, and stakeholder engagement to support sustainable and equitable gNBS implementation in the UAE.

## Introduction

Newborn screening is a modern public health capability, allowing early detection of congenital and metabolic disorders, which can lead to health complications, delayed developmental milestones, or even mortality if ignored. By enabling early identification of at-risk infants, Newborn Screening (NBS) Programs have significantly reduced infant morbidity and mortality, improved health outcomes, and lessened the burden on healthcare systems (1, 2).

The global newborn screening market is expected to grow at a 9.86% compound annual growth rate (CAGR) from $1.24 billion in 2023 to $2.16 billion by 2029, driven by government support, increased awareness, and technological advancements (3). Genomic Newborn Screening (gNBS), tandem mass spectrometry, and advanced DNA testing enhance the sensitivity and specificity of screening tests, enabling earlier and more accurate detection of genetic and metabolic abnormalities (4, 5). This paper shows how the UAE-Abu Dhabi is leading the way in newborn screening in the Middle East and North Africa (MENA) region, demonstrating its commitment to improving newborn health and reducing preventable diseases (6, 7); however, the effectiveness and delivery of these initiatives depend on healthcare professionals' implementation, promotion, and sustainability.

Several international initiatives have evaluated gNBS, including the BabySeq Project in the United States, population-based genomic pilot programs in Belgium, and implementation efforts within the United Kingdom National Health Service (5, 8–12). These studies have demonstrated the clinical utility of early genomic detection while also highlighting ethical, economic, and health-system challenges associated with scaling gNBS into routine care.

The UAE launched its National Neonatal Screening Program (NBS) in 1995, which covers over 40 disorders, including metabolic, endocrine, and genetic conditions such as phenylketonuria (PKU), congenital hypothyroidism, and sickle-cell disease (6). The program aims to prevent early health complications, improve neonatal outcomes, reduce diagnostic odyssey, and create a genetic disease database (13). However, barriers to its widespread implementation include a lack of awareness regarding the screening process, poor training, operational challenges to service delivery (14, 15), and inequalities in access to resources and healthcare infrastructure (16, 17). Therefore, understanding these issues is crucial for NBS's effectiveness.

The study investigates Abu Dhabi practitioners' attitudes, self-reported knowledge, and obstacles toward the expanded newborn screening program, offering valuable insights for policymakers, healthcare administrators, and practitioners. It highlights professional experiences, training, implementation, and policy support gaps, contributing to the global discourse on newborn screening, improved health outcomes, and the long-term sustainability of NBS programs.

### Method

The study employed a quantitative, cross-sectional design to assess healthcare professionals' self-reported knowledge, attitudes, and challenges regarding the expansion of gNBS in the UAE, using a piloted questionnaire distributed between June and January 2025, ensuring adequate time for participation.

### 2.1 Study population

The study targeted registered and active healthcare professionals in Abu Dhabi, including consultants, general practitioners, interns, residents, specialists, and allied healthcare professionals (including nurses, pharmacists, paramedics, and health educators), who were key stakeholders in implementing and sustaining newborn genomic screening programs in public and private healthcare facilities.

### 2.2 Sample size

The sample size was calculated using the formula for cross-sectional studies: *n* = *Z*^2^(1–*P*)/*d*^2^, where *Z* = 1.96 for a 95% confidence interval, expected prevalence of good gNBSknowledge among healthcare professionals, *P* = 0.8 as reported by Abeje (1), and *d* = 0.05 (margin of error). This yielded a minimum required sample of 243 participants.

### 2.3 Sampling strategy and data source

The study utilized the Shafafiya Portal, a centralized healthcare registry maintained by the Department of Health (DOH) Abu Dhabi (https://www.doh.gov.ae/en/shafafiya), to identify and invite eligible healthcare professionals. The database provided information on clinician licenses, specialties, facility names, and employment status, allowing stratification by urban and rural Abu Dhabi and pediatric experience. To reduce potential selection bias, random sampling was combined with chain sampling (snowballing), promoting broad participation across professions, experience levels, and healthcare settings (public/private, urban/rural). A stratified recruitment strategy further ensured a balanced representation of healthcare provider categories.

### 2.4 Recruitment and engagement

A multi-tiered recruitment strategy, employing random selection sampling and chain sampling (snowballing). Healthcare facilities were contacted for approval to distribute the survey. On-site visits were conducted to explain study objectives, obtain institutional approval, and provide printed surveys. Some institutions used internal survey distribution, allowing their HR departments to distribute printed surveys or email survey links to staff, and the snowball sampling method encouraged on-the-spot completion. Additionally, institutional collaboration was employed to increase participation.

### 2.5 Survey administration

The survey was offered in two formats: online via institutional email lists, WhatsApp groups, and professional networks, or printed during site visits. It remained open for 6 months to accommodate busy healthcare schedules, especially during the summer break, when many professionals were unavailable.

### 2.6 Survey instrument and measures

The survey, piloted among 50 healthcare professionals, was developed by adapting items and domains from previously published surveys assessing healthcare professionals' self-reported knowledge and attitudes toward newborn/genetic screening and genomic testing, and from implementation literature describing common barriers (1, 2, 18, 19). The draft instrument was reviewed for relevance to the Abu Dhabi context and piloted among healthcare professionals to improve clarity and face validity before full deployment. The pilot sample included physicians (consultants, residents), nurses, and allied health professionals working in pediatric and neonatal settings. Feedback was obtained regarding clarity, wording, and relevance of items. Minor modifications were made to improve question clarity and reduce ambiguity, particularly in the knowledge and ethical concern sections.

#### 2.6.1 Demographic information

Age, gender, professional role, work experience, institution type (government, private, clinic, etc.), work setting (urban vs. rural), and previous exposure to newborn screening technologies.

#### 2.6.2 Self-reported knowledge assessment

Familiarity with gNBS, understanding of gNBS applications and benefits, awareness of current UAE newborn screening protocols, and self-reported knowledge of TMS screening. Responses were categorized as good self-reported knowledge (know a lot, know some), or limited self-reported knowledge (know a little, do not know).

#### 2.6.3 Attitudes toward newborn genomic screening

Perceived necessity of gNBSin Abu Dhabi, interest in expanding genomic screening, concerns regarding NGS (including ethical issues, privacy concerns, psychological burden on families), opinions on screening criteria [including whole-genome sequencing (WGS) vs. next-generation sequencing panels], sequential vs. simultaneous screening with Tandem Mass Spectrometry (TMS).

#### 2.6.4 Barriers and challenges

Financial barriers (cost concerns and insurance coverage), technical challenges (need for advanced training and interpretation skills), healthcare system readiness (capacity for integrating genetic counseling services), ethical considerations (parental consent and data security concerns), implementation and policy considerations (preferred newborn screening technologies, policy recommendations for expanding genomic screening, identification of key disorders for inclusion in an expanded gNBS program, perceived role of nurses and midwives in sample collection from cord blood).

### 2.7 Ethical considerations

The study was approved by the UAE University Social Science Ethics Committee (ERSC 2024_4422), informed consent was obtained, and ethical guidelines outlined in the Declaration of Helsinki were adhered to. Participant confidentiality and anonymity were ensured, with the option to withdraw at any time. Data were securely stored on SurveyMonkey and were accessible only to the Principal Investigator.

### 2.8 Data analysis

Data were analyzed using R software (version 4.4.1). Categorical variables were summarized with frequencies and percentages. The level of self-reported knowledge about newborn genomic screening was categorized into two: good (comprising “*know a lot”* and “*know some”*) and limited (comprising “*know a little”* and “*I do not know”*). Pearson's Chi-squared test was used to assess associations between self-reported knowledge levels and participant characteristics, followed by binary logistic regression to adjust for confounders.

### 2.9 STROBE guidelines

This study followed the STROBE (Strengthening the Reporting of Observational Studies in Epidemiology) guidelines for cross-sectional studies, ensuring transparency and methodological rigor.

## 3 Results

### 3.1 Participant characteristics

A total of 362 participants, mostly females (61%) and 42% aged 30 years and below, were included in the study (Table 1). More than half of the participants (58%) had bachelor's degrees; others had either a master's degree (22%), a PhD (9%), or other qualifications (10%). The participants worked at various professional levels, including residents (21%), specialists (17%), general practitioners (11%), consultants (9.0%), interns (7%), and other professional titles (35.6%), with most of them working in urban areas (87%). A sizable number (43%) had academic experience, while 83% worked in pediatrics. Slightly more than half (55%) had experience in newborn screening, with 74% having, at most, 5 years of experience. Only 30% had experience with TMS screening programs.

**Table 1:** Participants' demographics and occupational characteristics (*N* = 362).

Characteristic	*N* (%)
Age (years)	
≤ 30	127 (42%)
31–35	51 (17%)
36–40	49 (16%)
41–45	24 (8.0%)
46–50	18 (6.0%)
> 50	32 (11%)
Gender	
Female	185 (61%)
Male	116 (39%)
Educational level	
Bachelor	176 (58%)
Masters	67 (22%)
PhD	27 (9.0%)
Other^a^	31 (10%)
Worked in academia (Yes)	128 (43%)
Professional title	
Consultant	27 (9.0%)
General practitioner	32 (11%)
Intern	21 (7.0%)
Resident	62 (21%)
Specialist	52 (17%)
Other^b^	107 (35.6%)
Worked with pediatric (Yes)	251 (83%)
Worked with newborn Screening (Yes)	166 (55%)
Years working with newborn Screening	
≤ 5	126 (74%)
6–10	25 (15%)
11–20	10 (5.8%)
>20	10 (5.8%)
Work location	
Rural	39 (13%)
Urban	262 (87%)
Experience with the TMS screening program	
No	138 (46%)
Yes	90 (30%)
I do not know	73 (24%)

^a^ Medical student, fellowship, Board Certified, diploma. ^b^ Dental assistant, paramedic, nurse, medical student, assistant nurse, pharmacist, health educator, Allied health, Genetic Counselor, Fellow, Director of Medical Education.

### 3.2 Self-reported knowledge of gNBS

Overall, 46% (95% CI: 40%−52%) of the participants reported having good self-reported knowledge of gNBS. The proportions of those with good gNBS self-reported knowledge were higher among academics (57% vs. 38%, *p* = 0.002), those working in urban areas (50% vs. 20%, *p* = 0.001), those who worked with newborn screening (58% vs. 31%, *p* < 0.001), those who had experience with TMS screening programs (72% vs. 34%, *p* < 0.001), those who have a higher interest in gNBS (60% vs. 15%, *p* < 0.001), and those willing to advocate for gNBS (50% vs. 12%, *p* < 0.001) (Table 2). Moreover, the prevalence of good gNBS self-reported knowledge was highest among interns/residents (57%), followed by specialists/consultants (50%), and general practitioners (32%). However, gNBS self-reported knowledge was not significantly associated with age, gender, education level, pediatric experience, or duration of working in newborn screening (*p* > 0.05).

**Table 2:** Factors associated with self-reported knowledge of genomic newborn screening (gNBS).

Characteristic	NGS knowledge level		
	Good knowledge (*n* = 133, 46%)	Limited knowledge (*n* = 155, 54%)	*p*-value
Age (years)			0.178
≤ 40	106 (48%)	113 (52%)	
> 40	27 (39%)	42 (61%)	
Gender			0.104
Female	76 (42%)	103 (58%)	
Male	57 (52%)	52 (48%)	
Educational level			0.400
Bachelor	83 (49%)	85 (51%)	
Postgraduate	38 (43%)	51 (57%)	
Other	12 (39%)	19 (61%)	
Worked in academia			0.002
No	62 (38%)	101 (62%)	
Yes	71 (57%)	54 (43%)	
Professional title			0.043
Trainees (interns/residents)	47 (57%)	35 (43%)	
General practitioner	9 (32%)	19 (68%)	
Specialists/consultants	38 (50%)	38 (50%)	
Other	28 (39%)	44 (61%)	
Worked with pediatric			0.186
No	18 (37%)	30 (63%)	
Yes	115 (48%)	125 (52%)	
Worked with newborn screening			< 0.001
No	39 (31%)	88 (69%)	
Yes	94 (58%)	67 (42%)	
Years working with newborn screening			0.528
≤ 5	63 (57%)	48 (43%)	
>5	25 (63%)	15 (37%)	
Work location			0.001
Rural	8 (22%)	29 (78%)	
Urban	125 (50%)	126 (50%)	
Experience with the TMS screening program			< 0.001
No	44 (34%)	85 (66%)	
Yes	63 (72%)	24 (28%)	
Interest in NGS			< 0.001
High	120 (60%)	80 (40%)	
Low	13 (15%)	75 (85%)	
Willingness to advocate for NGS			< 0.001
No	3 (12%)	21 (88%)	
Yes	120 (50%)	120 (50%)	

NGS: newborn genomic screening.

A multivariable model found that experience with the TMS screening program and interest in gNBS were independently associated with gNBS self-reported knowledge level (Table 3). Those who had experience with the TMS screening program (OR = 2.92, 95% CI = 1.16–7.56, *p* = 0.024) and those with a higher interest in gNBS (OR = 10.3, 3.74–33.5, *p* < 0.001) were significantly more likely to have good self-reported knowledge of gNBS.

**Table 3:** Univariate and multivariable logistic regression analysis for factors associated with good knowledge of newborn genomic screening.

Characteristic	cOR	95% CI	*p*-value	aOR	95% CI	*p*-value
Worked in academia						
No	1.00	—		1.00	—	
Yes	2.14	1.34, 3.46	**0.002**	1.02	0.46, 2.22	>0.900
Professional title						
Trainees (interns/residents)	1.00	—		1.00	—	
General practitioner	0.35	0.14, 0.85	**0.024**	0.34	0.08, 1.40	0.140
Specialists/consultants	0.74	0.40, 1.39	0.400	0.42	0.15, 1.14	0.094
Other	0.47	0.25, 0.90	**0.023**	0.53	0.17, 1.56	0.200
Worked with newborn screening						
No	1.00	—		1.00	—	
Yes	3.17	1.95, 5.21	**< 0.001**	2.29	0.96, 5.52	0.062
Work location						
Rural	1.00	—		1.00	—	
Urban	3.6	1.65, 8.71	**0.002**	2.14	0.62, 8.06	0.200
Experience with the TMS screening program						
No	1.00	—		1.00	—	
Yes	5.07	2.83, 9.32	**< 0.001**	2.92	1.16, 7.56	**0.024**
Interest in NGS						
Low	1.00	—		1.00	—	
High	8.65	4.64, 17.3	**< 0.001**	10.3	3.74, 33.5	**< 0.001**
Willingness to advocate for NGS						
No	1.00	—		1.00	—	
Yes	7	2.34, 30.2	**0.002**	4.97	1.05, 37.2	0.067

NGS: newborn genomic screening. cOR: Crude odds ratio. aOR: Adjusted odds ratio. Bold values indicate statistically significant associations.

### 3.3 Attitudes toward gNBS and ethical concerns

The majority of participants have some interest in gNBS (Supplementary Figure 1), with specialists/consultants (75%), followed by general practitioners (64%), and trainees (61%). Many participants recommended including SCID (45%), SMA (44%), and DMD (40%) in the expanded gNBS program (Supplementary Figure 2).

Many participants cited potential harm from misunderstanding and misinterpretation of genetic information (38%), privacy (36%), and the psychological burden of carrying information about adult morbidity risk (34%) as major ethical issues in gNBS (Supplementary Figure 3). Additional free-text responses highlighted concerns related to equity and access to testing, potential insurance discrimination based on genetic information, stigmatization of affected families, inclusivity in screening eligibility, and the ethical implications of false-negative results. Some believed that expanded gNBS is unsuitable due to its low popularity and high costs (28%), lack of uniformity in genetic diseases and pathogenic genes (20%), and ethical and privacy issues (17%), among others (Supplementary Figure 4).

Most participants believed that gNBS offers significant advantages, including its ability to extend screening to diseases not suitable for biochemical analysis or that lack reliable biomarkers (39%), clarifying ambiguous results and guiding accurate medication (25%), and determining mutation sources at the molecular level (21%) (Supplementary Figure 5).

PCR combined with next-generation sequencing panels (25%) and whole-genome sequencing (25%) were the most popular screening technologies among the participants. However, 30% of participants were unaware of these technologies (Supplementary Figure 6). Key factors for choosing screening technology included high throughput, large data volume, and accuracy (39%), cost and price (31%), simple operation, localization detection, and quality control (29%), and advanced technology (27%), among others (Supplementary Figure 7).

The majority of respondents (42%) said all newborns and those with positive MS/MS screening results (29%) should undergo gNBS (Supplementary Figure 8).

### 3.4 Resources needed for the clinical application of gNBS

Regarding the resources needed for the clinical application of gNBS, 63% of participants mentioned knowledge of neonatal genetic diseases, such as diagnosis, treatment, and intervention. Other suggested resources included abilities in report interpretation and genetic counseling (40%), and policies, regulations, technical specifications, and expert consensus (36%) (Supplementary Figure 9).

Furthermore, 85% of participants agreed that nurses and midwives should be upskilled in cord blood sample collection (Supplementary Figure 10).

Table 4 presents participants' responses on the necessity of gNBS in Abu Dhabi, major concerns, screening principles, the application modes of NGS and TMS, capabilities improvement, the main reasons for promoting expanded gNBS, and reasonable prices for gNBS.

**Table 4:** Attitudes and perceptions toward NGS among participants (*N* = 362).

Questions and responses	Response, *N* (%)
Do you think it is necessary to carry out NGS in Abu Dhabi?	
Very much necessary	178 (61%)
Somewhat necessary	91 (31%)
Unnecessary	6 (2.1%)
I do not know	15 (5.2%)
What is your biggest concern about NGS?	
As a screening technology, it is unsatisfactory in terms of popularity, reporting time, and high cost.	31 (11%)
Great challenge for clinical counseling ability because of too much genetic information.	57 (20%)
Lack of treatment interventions for screening diseases.	70 (24%)
Too much genetic information may cause ethical, counseling, and psychological burden problems.	96 (33%)
I do not know	35 (12%)
What is your view about the principles for screening disease types, pathogenic genes, and mutations?	
Both complement each other, and Tandem Mass Spectrometry (MS/MS) screening cannot be completely replaced.	63 (23%)
Including at least 90% of the diseases by Tandem Mass Spectrometry (MS/MS) screening.	9 (3.3%)
It only includes pathogenic and like pathogenic mutations, while excluding Variants of Uncertain Significance (VUS).	11 (4.0%)
Some serious genetic diseases have high incidence and can be intervened or treated.	88 (32%)
The more diseases screened, the better.	73 (26%)
I do not know	32 (12%)
What do you think about the reasonable application mode of NGS and Tandem Mass Spectrometry (MS/MS) screening?	
Independent screening mode: both are completely independent, and the results are compared and summarized.	61 (22%)
It doesn't matter: As two independent methods, they are independent management	16 (5.8%)
Sequential screening mode: traditional screening is carried out first, and NGS is carried out for suspected positive	96 (35%)
Unite screening mode: Both test at the same time, summarize the results, and recall for diagnosis	39 (14%)
I do not know	65 (23%)
How do you want to improve the above capabilities?	
Academic conferences and education projects	149 (53%)
CME online courses	32 (11%)
Network platform	18 (6.5%)
Symposium, case discussion, multicenter exchange	68 (24%)
Other^a^	12 (4.3%)
What do you think are the most important technical problems to be solved before the application of NGS?	
Clear that it is screening technology, not diagnostic technology	96 (35%)
Current technical conditions are sufficient to meet the clinical needs	13 (4.7%)
The relationship with traditional screening	27 (9.7%)
The scope and ability of genetic counseling	74 (27%)
The screening technology	59 (21%)
Other (please specify)	9 (3.2%)
What do you think is the main reason that hinders the promotion of expanded NGS?	
Healthcare professionals are reluctant to promote because of lack of abilities	38 (14%)
The cost is too expensive	66 (25%)
The diseases which are screened can't be effectively treated and intervened	66 (25%)
The genetic counseling ability and relevant supporting policies cannot be met	29 (11%)
The technology is not mature and cannot be used for large-scale screening	34 (13%)
Other^b^	3 (1.1%)
I do not know	33 (12%)
What do you think is a reasonable price for NGS?	
AED < 300	41 (15%)
AED 300–600	48 (18%)
AED 700–1200	41 (15%)
> AED 1200	7 (2.6%)
Should be covered by insurance	132 (49%)

^a^ All of the above, some of the above, intensive NGS training workshops, utilizing visual aids/short videos. ^b^ Insufficient information about the technology, lack of knowledge of NGS and logistics.

## 4 Discussion

Our study surveyed 362 healthcare professionals in Abu Dhabi to assess their self-reported knowledge, attitudes, and challenges regarding the expansion of gNBS, with 83% having pediatric experience and 58% having worked in newborn screening programs. Self-reported knowledge of gNBS varied, with 46% having good self-reported knowledge and the remaining having limited awareness. Factors associated with higher gNBS self-reported knowledge included working in academia, experience in newborn screening, urban practice settings, and prior experience with TMS. Barriers to gNBS implementation included cost concerns, lack of unified genetic screening guidelines, and ethical/privacy concerns. Most believed gNBS is necessary in Abu Dhabi, while 70% expressed interest in its implementation. The top recommended conditions for expanded gNBS programs were SCID, DMD, and SMA. Key challenges included misinterpretation of genetic information, ethical concerns, and privacy risks.

Our findings are broadly consistent with international studies examining healthcare professionals' perspectives on genomic and expanded newborn screening. Similar surveys have reported variable genomic literacy, strong interest in integration, and concerns related to cost, ethical complexity, and workforce preparedness (2, 18, 19). The alignment of our results with these studies suggests that the challenges identified in Abu Dhabi reflect broader global implementation dynamics rather than isolated local barriers.

A study by Boland et al. (2) highlights that exposure to gNBS testing and education significantly impacts healthcare professionals' knowledge levels. These findings align with our observation that participants with a higher interest in gNBS or experience in related fields exhibit better knowledge. A recent study by Nurchis et al. (3) supports our findings that professional titles (e.g., interns/residents) are associated with better knowledge, possibly due to more encounters with diagnostic technologies. Furthermore, the UAE's genomic education curricula identified a gap in the coverage of genomic topics at the undergraduate and postgraduate levels (4).

The UAE's diverse healthcare workforce, comprising expatriate professionals from various countries, including South Asia, the Middle East, Africa, Europe, and North America, may influence attitudes toward expanded gNBS due to their diverse training backgrounds, genomic technology experiences, and cultural attitudes toward genetic screening (10). This diversity may influence how healthcare professionals perceive ethical concerns, feasibility, and utility of gNBS. Future research should explore how cultural and educational backgrounds influence perceptions and readiness to implement genomic innovations in a diverse healthcare system like the UAE.

The analysis of the BabySeq Project's genomic sequencing (20) confirms the association between experience with newborn screening programs and higher gNBS knowledge. Similarly, our study found that prior experience with TMS is associated with better healthcare professional knowledge.

Healthcare professionals in our cohort recommended the inclusion of SCID, DMD, and SMA in expanded gNBS programs in the UAE, aligning with local priorities identified by the National Reference Laboratory and with global newborn screening initiatives (11, 13, 21). The prioritization of SCID, SMA, and DMD aligns with established screening principles emphasizing disease severity, early onset, and clinical actionability (22). Early detection of SCID significantly improves survival through timely intervention (23). Similarly, presymptomatic treatment of SMA following newborn screening leads to substantially improved outcomes (24). Although DMD presents additional challenges, long-standing screening experience and evolving therapeutic options support arguments for early identification (25). These findings suggest that healthcare professionals favor inclusion of conditions with clear therapeutic or management benefits.

Ethical implications of genetic testing have been extensively studied, with studies showing increased psychological distress (9, 26, 27) and potentially affecting family members due to shared genetic traits (28). Our cohort selected misunderstanding of genetic information, privacy of genetic information, and psychological burden as the main ethical concerns of gNBS. Addressing these challenges requires implementing policies and enhancing literacy, with genetic counselors and Obstetricians-Gynecologists at the forefront in promoting genetic literacy in the community (8, 12, 29).

The reasons cited by the healthcare providers in the UAE are their hesitation to expand gNBS due to high costs and a lack of unified genetic targets, as noted in the literature. Nevertheless, in our cohort, the cost was cited as a challenge. However, studies show that sequencing costs are decreasing, making gNBS more feasible (19).

On the other hand, healthcare professionals believe that gNBS should include diseases with high penetrance, early treatment, and strong genotype-phenotype correlation. Many genetic diseases do not meet these criteria, complicating the selection of standardized screening panels and introducing technical challenges (19, 21, 30).

Despite these challenges, healthcare professionals highlight the benefits of gNBS, including expanding the scope of disease, clarifying ambiguous results, and pinpointing mutations at the molecular level, supported by genomic medicine studies and advancements (9, 11, 26–28).

The perception that all newborns (41%) or those with positive MS/MS screening results (33%) should undergo next-generation sequencing (NGS) is consistent with current research and pilot studies. For example, Belgium successfully identified 71 clinical diagnoses in 3,847 newborns using NGS, some of which were overlooked by TMS (8). Moreover, in the USA, GUARDIAN (Genomic Uniform-screening Against Rare Diseases in All Newborns) achieved a screen-positive rate of 3.7% (12).

Healthcare professionals prioritize resources for gNBS implementation, including knowledge of neonatal genetic diseases, report interpretation, genetic counseling, policies, regulations, and expert consensus, aligning with global studies and initiatives (5, 14).

Our cohort strongly supports capacity building for nurses and midwives to improve their skills in cord blood collection, despite research showing that many nurses lack adequate knowledge, with 86% demonstrating poor knowledge before training interventions (29).

Finally, healthcare providers are using academic conferences, online CME courses, collaborative platforms, and case-based learning to enhance their capabilities for gNBS. This aligns with a qualitative study guided by the Action, Actor, Context, Target, and Time framework, with Key informant perspectives on implementing gNBS (19). In this study, educating health professionals was ranked first in the implementation process (stage 0) (19). On the other hand, Chinese multicenter studies steered the importance of case discussions to improve diagnostic accuracy and refine screening panels based on regional disease prevalence (30).

This study has several limitations: response bias, lack of exploration of religion, cultural, or psychosocial factors, a broad in-scope survey instrument, and the absence of Likert-scale items may have restricted the ability to measure participant attitudes toward gNBS. Future research should include in-depth interviews or focus groups to capture underlying beliefs and values. Although the sample size (*n* = 362) met the calculated requirement, the study was conducted in a single Emirate, which may limit generalizability to other regions of the UAE. Additionally, the study may under-represent certain healthcare professionals, especially those in rural areas, due to differences in genomic screening technologies.

### 4.1 Implications for policy and practice

The findings of this study have short- and long-term implications for genomic newborn screening implementation in the UAE. In the short term, structured training programs, clear national guidelines, and strengthened genetic counseling capacity are essential to address knowledge gaps and ethical concerns identified by healthcare professionals. In the longer term, development of transparent, evidence-based frameworks for condition selection, cost-effectiveness evaluation, and regulatory oversight will be critical to ensure sustainable and equitable integration of genomic newborn screening into routine neonatal care. Engagement of key stakeholders, including policymakers, clinicians, and community representatives, will be important to support responsible expansion efforts.

## 5 Conclusion

This study highlights variability in self-reported genomic knowledge among healthcare professionals in Abu Dhabi and identifies cost, ethical considerations, and guideline development as key perceived challenges to expanding genomic newborn screening. The findings suggest that strengthening training initiatives, clarifying policy frameworks, and enhancing genetic counseling capacity may support future implementation efforts. Further research involving broader stakeholder groups will be important to inform responsible and sustainable expansion of genomic newborn screening in the UAE.

## Data Availability

The raw data supporting the conclusions of this article will be made available by the authors, without undue reservation.

## References

[1] Abeje TA. The perspectives of health professionals on neonatal genetic screening. Curr Pharmacogenomics Pers Med. (2022) 19:31–9. doi: 10.2174/1875692119666220225140848

[2] Boland PM Ruth K Matro JM Rainey KL Fang CY Wong YN . Genetic counselors'(GC) knowledge, awareness, understanding of clinical next-generation sequencing (NGS) genomic testing. Clin Genet. (2015) 88:565–72. doi: 10.1111/cge.12555 25523111 PMC4474774

[3] Nurchis MC Altamura G Raspolini GM Capobianco E Salmasi L Damiani G. Health professionals' preferences for next-generation sequencing in the diagnosis of suspected genetic disorders in the paediatric population. J Pers Med. (2025) 15:25. doi: 10.3390/jpm15010025 39852217 PMC11766785

[4] Rahma AT Ahmed LA Elsheik M Elbarazi I Ali BR Patrinos GP . Mapping the educational environment of genomics and pharmacogenomics in the United Arab Emirates: a mixed-methods triangulated design. OMICS. (2021) 25:285–93. doi: 10.1089/omi.2021.0029 33904793

[5] Ceyhan-Birsoy O Murry JB Machini K Lebo MS Yu TW Fayer S . Interpretation of genomic sequencing results in healthy and ill newborns: results from the BabySeq project. Am J Hum Genet. (2019) 104:76–93. doi: 10.1016/j.ajhg.2018.11.016 30609409 PMC6323417

[6] Gaviglio A Lasarev M Sheller R Singh S Baker M. Newborn screening for severe combined immunodeficiency: lessons learned from screening and follow-up of the preterm newborn population. Int J Neonatal Screen. (2023) 9:68. doi: 10.3390/ijns9040068 38132827 PMC10744167

[7] Bizzari S Nair P Hana S Deepthi A Al-Ali MT Al-Gazali L . Spectrum of genetic disorders and gene variants in the United Arab Emirates national population: insights from the CTGA database. Front Genet. (2023) 14:1177204. doi: 10.3389/fgene.2023.1177204 37214420 PMC10194840

[8] Boemer F Hovhannesyan K Piazzon F Minner F Mni M Jacquemin V . Population-based, first-tier genomic newborn screening in the maternity ward. Nat Med. (2025) 31:1339–50. doi: 10.1038/s41591-024-03465-x 39875687 PMC12003153

[9] van Campen JC Sollars ESA Thomas RC Bartlett CM Milano A Parker MD . Next generation sequencing in newborn screening in the United Kingdom National Health Service. Int J Neonatal Screen. (2019) 5:40. doi: 10.3390/ijns5040040 31844782 PMC6914376

[10] Remec ZI Trebusak Podkrajsek K Repic Lampret B Kovac J Groselj U Tesovnik T . Next-generation sequencing in newborn screening: a review of current state. Front Genet. (2021) 12:662254. doi: 10.3389/fgene.2021.662254 34122514 PMC8188483

[11] Bick D Ahmed A Deen D Ferlini A Garnier N Kasperaviciute D . Newborn screening by genomic sequencing: opportunities and challenges. Int J Neonatal Screen. (2022) 8:40. doi: 10.3390/ijns8030040 35892470 PMC9326745

[12] Ziegler A Koval-Burt C Kay DM Suchy SF Begtrup A Langley KG . Expanded newborn screening using genome sequencing for early actionable conditions. JAMA. (2025) 333:232–40. doi: 10.1001/jama.2024.27289 39446378 PMC11503470

[13] Skrinska V Khneisser I Schielen P Loeber G. Introducing and expanding newborn screening in the MENA region. Int J Neonatal Screen. (2020) 6:12. doi: 10.3390/ijns6010012 33073010 PMC7422969

[14] Chen R Pearson V Suebkinorn O Bulto LN Pincha Baduge M Anderson A . Impact of genetic risk information for cardiovascular disease on behavioural changes, psychological responses and risk factor modification: a systematic review. Eur J Prev Cardiol. (2024) 3:327–37. doi: 10.1093/eurjpc/zwae362 39489494

[15] Heshka JT Palleschi C Howley H Wilson B Wells PS. A systematic review of perceived risks, psychological and behavioral impacts of genetic testing. Genet Med. (2008) 10:19–32. doi: 10.1097/GIM.0b013e31815f524f 18197053

[16] Ahn W-k Perricone AM. Impacts of learning one's own genetic susceptibility to mental disorders. Curr Dir Psychol Sci. (2023) 32:42–8. doi: 10.1177/09637214221127225

[17] Kudiabor H. ‘Anonymous' genetic databases vulnerable to privacy leaks. Nature. (2024) 634:764–5. doi: 10.1038/d41586-024-03236-1 39402295

[18] Wu X Yang Y Zhou L Long W Yu B. Are we ready for newborn genetic screening? A cross-sectional survey of healthcare professionals in Southeast China. Front Pediatr. (2022) 10:875229. doi: 10.3389/fped.2022.875229 35601442 PMC9120836

[19] Tutty E Archibald AD Downie L Gaff C Lunke S Vears DF . Key informant perspectives on implementing genomic newborn screening: a qualitative study guided by the action, actor, context, target, time framework. Eur J Hum Genet. (2024) 32:1599–605. doi: 10.1038/s41431-024-01650-7 38907005 PMC11606939

[20] The Lancet. Genomic newborn screening: current concerns and challenges. Lancet. (2023) 402:265. doi: 10.1016/S0140-6736(23)01513-1 37481265

[21] Lincoln SE Hambuch T Zook JM Bristow SL Hatchell K Truty R . One in seven pathogenic variants can be challenging to detect by NGS: an analysis of 450,000 patients with implications for clinical sensitivity and genetic test implementation. Genet Med. (2021) 23:1673–80. doi: 10.1038/s41436-021-01187-w 34007000 PMC8460443

[22] Andermann A Blancquaert I Beauchamp S Déry V. Revisiting Wilson and Jungner in the genomic age: a review of screening criteria over the past 40 years. Bull World Health Organ. (2008) 86:317–9. doi: 10.2471/BLT.07.050112 18438522 PMC2647421

[23] Kwan A Abraham RS Currier R Brower A Andruszewski K Abbott JK . Newborn screening for severe combined immunodeficiency in 11 screening programs in the United States. JAMA. (2014) 312:729–38. doi: 10.1001/jama.2014.9132 25138334 PMC4492158

[24] Glascock J Sampson J Haidet-Phillips A Connolly A Darras B Day J . Treatment algorithm for infants diagnosed with spinal muscular atrophy through newborn screening. J Neuromuscl Dis. (2018) 5:145–58. doi: 10.3233/JND-180304 PMC6004919 29614695

[25] Moat SJ Bradley DM Salmon R Clarke A Hartley L. Newborn bloodspot screening for Duchenne muscular dystrophy: 21 years experience in Wales (UK). Eur J Hum Genet. (2013) 21:1049–53. doi: 10.1038/ejhg.2012.301 23340516 PMC3778339

[26] Boemer F Fasquelle C d'Otreppe S Josse C Dideberg V Segers K . A next-generation newborn screening pilot study: NGS on dried blood spots detects causal mutations in patients with inherited metabolic diseases. Sci Rep. (2017) 7:17641. doi: 10.1038/s41598-017-18038-x 29247206 PMC5732277

[27] Veldman A Kiewiet MBG Heiner-Fokkema MR Nelen MR Sinke RJ Sikkema-Raddatz B . Towards next-generation sequencing (NGS)-based newborn screening: a technical study to prepare for the challenges ahead. Int J Neonatal Screen. (2022) 8:17. doi: 10.3390/ijns8010017 35323196 PMC8949100

[28] Jiang S Wang H Gu Y. Genome sequencing for newborn screening—an effective approach for tackling rare diseases. JAMA Netw Open. (2023) 6:e2331141. doi: 10.1001/jamanetworkopen.2023.31141 37656463

[29] Mohammed HS El Sayed HA. Knowledge and attitude of maternity nurses regarding cord blood collection and stem cells: an educational intervention. J Nurs Educ Pract. (2015) 5:58. doi: 10.5430/jnep.v5n4p58

[30] Yang RL Qian GL Wu DW Miao JK Yang X Wu BQ . A multicenter prospective study of next-generation sequencing-based newborn screening for monogenic genetic diseases in China. World J Pediatr. (2023) 19:663–73. doi: 10.1007/s12519-022-00670-x 36847978 PMC10258179

