# Supplementary material for: Healthcare professionals' perspectives on expanding newborn genomic screening in the emirate of Abu Dhabi, United Arab Emirates: insights from a cross-sectional study
Source: Front Public Health. 2026 Jun 3;14:1769908. doi: 10.3389/fpubh.2026.1769908 (PMC13272335; doi:10.3389/fpubh.2026.1769908)
Supplement: Supplementary file 1 [file Data_Sheet_1.docx]

Supplementary Material

**Figure 1: Participants' level of interest in newborn genetic screening by professional title**

**Figure 2: Disorders recommended by the participants to be included in the expanded genomic newborn screening program in the UAE**

Other: Phenylketonuria, Trisomy (21,18,13), fragile x syndrome, Klinefelter syndrome, turner syndrome, thalassemia, G6PD deficiency, cystic fibrosis, Thalassemia, SCA, other hemoglobinopathies and metabolic disorders that have a defined successful medical intervention and treatment

**Figure 3: What do you think are the main ethical problems of newborn genomic screening?**

Other: Equity and accessibility issues, insurance discrimination after acquiring genetic information, stigmatization, inclusivity, false negatives

**Figure 4: If you think it is not suitable to carry out expanded NGS now, what are the main reasons?**

**Figure 5: What do you think is the biggest advantage of NGS?**

**Figure 6: Which technology do you think is suitable for screening in the UAE?**

Other: Microarray

**Figure 7: What are the main reasons when choosing the suitable screening technology?**

**Figure 8: At present, what do you think is the suitable population for NGS?**

**Figure 9: What resources do you think are needed for clinical application of NGS?**

Other: I don't know

**Figure 10: Should nurses and midwives be upskilled to collect samples from cord blood? (N=253)**
